# Leukadherin-1 ameliorates endothelial barrier damage mediated by neutrophils from critically ill patients

**DOI:** 10.1186/s40560-018-0289-5

**Published:** 2018-03-15

**Authors:** Catherine M. Dickinson, Brian W. LeBlanc, Muhammad M. Edhi, Daithi S. Heffernan, Mohd. Hafeez Faridi, Vineet Gupta, William G. Cioffi, Xian O’Brien, Jonathan S. Reichner

**Affiliations:** 1 Rhode Island Hospital Division of Surgical Research, Department of Surgery, Alpert Medical School of Brown University, Providence, RI USA; 2 College of Pharmacy, Chicago State University, Chicago, IL USA; 3 Rush University Medical Center, Chicago, IL USA

**Keywords:** Neutrophil, Endothelium, Barrier function, Sepsis, Trauma, Leukadherin-1

## Abstract

**Background:**

Multi-organ failure occurs during critical illness and is mediated in part by destructive neutrophil-to-endothelial interactions. The β2 integrin receptor, CR3 (complement receptor 3; Mac-1; CD11b/CD18), which binds endothelial intercellular adhesion molecule-1 (ICAM-1), plays a key role in promoting the adhesion of activated neutrophils to inflamed endothelia which, when prolonged and excessive, can cause vascular damage. Leukadherin-1 (LA-1) is a small molecule allosteric activator of CR3 and has been shown to promote adhesion of blood neutrophils to inflamed endothelium and restrict tissue infiltration. Therefore, LA-1 offers a novel mechanism of anti-inflammatory action by activation, rather than inhibition, of the neutrophil CR3 integrin. However, whether promotion of neutrophil-to-endothelial interaction by this novel therapeutic is of benefit or detriment to endothelial barrier function is not known.

**Methods:**

Critically ill septic and trauma patients were prospectively enrolled from the surgical and the trauma ICU. Blood was collected from these patients and healthy volunteers. Neutrophils were isolated by dextran sedimentation and adhered to TNF-α (tumor necrosis factor-α)-activated human umbilical vein endothelial (HUVEC) monolayers in the presence or absence of fMLP (formylmethionine-leucine-phenylalanine) and/or LA-1. Electric cell-substrate impedance sensing (ECIS) and exposure of underlying collagen were used to quantify endothelial barrier function and permeability.

**Results:**

Neutrophils from critically ill trauma and septic patients caused similar degrees of endothelial barrier disruption which exceeded that caused by cells obtained from healthy controls both kinetically and quantitatively. LA-1 protected barrier function in the absence and presence of fMLP which served as a secondary stimulant to cause maximal loss of barrier function. LA-1 protection was also observed by quantifying collagen exposure underlying endothelial cells challenged with fMLP-stimulated neutrophils. LA-1 treatment resulted in decreased migration dynamics of neutrophils crawling on an endothelial monolayer with reduced speed (μm/s = 0.25 ± 0.01 vs. 0.06 ± 0.01, *p* < 0.05), path length (μm = 199.5 ± 14.3 vs. 42.1 ± 13.0, *p* < 0.05), and displacement (μm = 65.2 ± 4.7 vs. 10.4 ± 1.3; *p* < 0.05).

**Conclusion:**

Neutrophils from patients with trauma or sepsis cause endothelial barrier disruption to a similar extent relative to each other. The CR3 agonist LA-1 protects endothelial barrier function from damage caused by neutrophils obtained from both populations of critically ill patients even when exposed to secondary stimulation.

**Electronic supplementary material:**

The online version of this article (10.1186/s40560-018-0289-5) contains supplementary material, which is available to authorized users.

## Background

The neutrophilic response to a localized injury or infection includes a transient interaction with vascular endothelial cells as peripheral blood neutrophils cross from the circulation into an afflicted tissue [1]. Because of the transient interaction between the leukocyte and endothelium, damage to the vasculature is minimal. Pathology can result when the inflammatory response becomes abnormally persistent due to a failure of resolution [2]. Nonresolving inflammation is a contributing factor to such diseases as chronic obstructive pulmonary disease, obesity, atherosclerosis, multiple sclerosis, and inflammatory bowel disease. When an injury or infection is severe, such as in the critically ill post-trauma or septic patient, systemic hyperactivation of both endothelial cells and neutrophils results in leukocyte-mediated vascular damage which in turn is a contributing factor to multi-system organ failure [3, 4]. Indeed, even if a patient survives the initial critical illness, morbidity and mortality can be driven by a protracted course of endothelial leak and end-organ failure [5]. Taken together, there is significant clinical evidence for a mechanistic role of endothelial damage resulting to organ dysfunction.

Leukocyte integrins, including CR3 (complement receptor 3; Mac-1; CD11b/CD18; αMβ2), are essential for the migration and recruitment of immune cells such as neutrophils, macrophages, and dendritic cells to damaged or infected organs [6, 7]. Integrins mediate the attachment of neutrophils to the vasculature and resist detachment by shear flow sufficiently to permit diapedesis. Because of the central role of CR3 in the infiltration of neutrophils into the sites of injury or infection, it is reasonable to hypothesize that blocking this receptor-ligand interaction may dampen the damaging consequences of the systemic inflammatory response that accompanies critical illness. However, integrin antagonists have failed to provide a clinical benefit to the septic or injured patient [8]. Alternatively, a small molecule allosteric agonist of the CR3 integrin termed leukadherin-1 (LA-1) has shown anti-inflammatory bioactivity in small animal models and in ex vivo experiments. LA-1 is an allosteric agonist because it activates the integrin by binding to a site distal from the site of ligand binding [9]. Leukadherin-1 suppresses neutrophilic infiltration into an inflamed or infected site but does so through a counterintuitive mechanism. LA-1 increases the CD11b-dependent cell adhesion to CR3 ligands including ICAM-1, decreases neutrophil rolling along TNF-α-stimulated venules, and increased the number of adherent cells [9, 10]. Therefore, LA-1 promotes the margination and adhesion of neutrophils to the endothelial lining of blood vessels without allowing subsequent extravasation. Extension of the frequency and temporal interaction between neutrophils and endothelial cells supports the hypothesis that endothelial barrier function may be compromised.

Electric cell impedance sensing (ECIS) is a quantifiable, real-time measure of endothelial barrier function [11]. Using ECIS, we previously reported that neutrophils from septic patients with ARDS have a more deleterious effect on endothelial barrier function than neutrophils from patients with sepsis but without ARDS or ex vivo activated neutrophils isolated from healthy donors [12]. Given the potential of LA-1 for therapeutic control of a damaging inflammatory response, understanding the mechanism of action of this molecule is of interest. The effect of LA-1 on increasing neutrophil margination raises the cautionary question as to whether LA-1 can exacerbate endothelial damage and thereby increase leak. This concern is rooted in the deleterious effect of activated neutrophils on endothelial barrier function which could potentially be worsened by increasing neutrophil adhesion to the endothelial target [8]. The current study has two primary objectives: first, to determine the effect LA-1 treatment of neutrophils on endothelial barrier function, and second, to conduct these studies using human neutrophils isolated from critically ill trauma and sepsis patients in the ICU. These objectives are designed to test the hypothesis that LA-1 treatment of activated human neutrophils may worsen endothelial barrier function.

## Methods

### Reagents

Lyophilized thrombin from human plasma, l-cysteine, fMLP, Histopaque 1077, and dextran (~ 80–120 kDa molecular mass) were obtained from Sigma-Aldrich Life Sciences (St. Louis, MO). Rat-tail type I collagen was obtained from BD Biosciences (Bedford, MA). Recombinant human TNF-α was obtained from R&D Systems (Minneapolis, MN). Human umbilical vein endothelial cells (HUVEC), trypsin, and endothelial growth medium (EGM-2), containing SingleQuots supplements®, were purchased from Lonza (Walkersville, MD). LA-1 was purchased from Chembridge Corp. (Cat. No. 5679982, San Diego, CA). Electric cell-substrate impedance sensing (ECIS) cultureware electrode arrays (8W10E+) and 16-well array station were obtained from Applied BioPhysics (Troy, NY). All reagents used contained < 0.1 pg/mL endotoxin as determined by Limulus amebocyte lysate screening (Lonza).

### Patient enrollment

Study approval was obtained from the Institutional Review Board of Rhode Island Hospital. Written informed consent to participate was provided by the patients or their surrogates. Critically ill septic patients and trauma patients were prospectively enrolled from the surgical ICU and the trauma ICU. Septic patients were identified as those with two or more systemic inflammatory response syndrome criteria with a source of infection. Patients were diagnosed with sepsis based on clinical evidence or microbiological data. Trauma patients were defined as patients with injuries severe enough to warrant ICU admission. Patients were enrolled within 24 h of their diagnosis or admission.

The patient charts were reviewed for demographics, laboratory values, and vitals. In addition, charts and microbiologic data were reviewed to identify the source of sepsis and the injuries that had been sustained. Data collected was used to calculate the Acute Physiology and Chronic Health Evaluation II (APACHE-II) score and Sequential Organ Failure Assessment (SOFA) score for the worst values in the 24 h after admission. Patients were enrolled if they were 18 years of age or older and were excluded if they had a massive blood transfusion of 4 or more units or were on any medications known to alter neutrophil functioning such as high-dose corticosteroids or chemotherapy.

### Neutrophil isolation

Up to 15 mL of blood were collected in sterile tubes containing EDTA and were processed within 30 min of collection without storage. Neutrophils were isolated from whole blood using gradient centrifugation on Histopaque. Sedimentation of erythrocytes was performed using 3% dextran, and the neutrophil-rich supernatant then underwent hypotonic lysis of residual erythrocytes, yielding a > 95% pure neutrophil population of > 90% viability by trypan dye exclusion. Neutrophils were resuspended in Hanks balanced salt solution without calcium and magnesium and counted.

### Leukadherin-1 treatment

Purified neutrophils were pre-treated with 15 μM of LA-1 on ice for 30 min prior to their use in barrier function assays, transmigration assays, or microscopy. A 0.5% DMSO solution was used as a concentration-matched vehicle control.

### Endothelial cell culture

HUVECS were grown to confluence on gelatin-coated flasks at 37 °C with 5% CO_2_ in EGM-2 medium. Cells within the first five passages were trypsinized once confluent. They were then counted and used in barrier function assays, microscopy, and transmigration assays.

### Measurement of endothelial barrier function

Transendothelial electrical resistance was measured in real time allowing determination of endothelial barrier function as it provides resistance to the current flow across the electrode (Applied Biophysics, Troy, NY). Wells of sterile 8W10E+ electrode arrays were coated with 40 μg/mL type I collagen and then plated with HUVEC and grown to confluence. The endothelial cells were then incubated with recombinant human TNF-α (20 ng/mL) for a period of 3 h prior to the addition of neutrophils to mimic the activated endothelium that would occur in vivo. Resistance measurements were collected at 4000 Hz. Isolated neutrophils either pre-treated with LA-1, vehicle control, or no pre-treatment were added (1 × 10^6^ cells per well). fMLP was added to a subset of wells (final concentration 10^−6^ M) 30 min after neutrophil addition to ensure the neutrophils had settled to the bottom of the well. Monolayer resistance was recorded over 4 h in 4-s intervals in the incubator (37 °C, 5% CO_2_). Thrombin (0.8 units/mL) served as a positive control, and EGM-2 medium served as a negative control.

### Endothelial permeability imaging

HUVECs were plated on biotinylated type I collagen-coated delta T dishes and allowed to grow to confluence and TNF-α-activated prior to neutrophil addition [13]. Neutrophils were either untreated or treated with 15 μM LA-1 or DMSO vehicle control and then added to HUVEC monolayers, stimulated with 10^−6^ M fMLP, and incubated for 40 min. The media was then aspirated and the monolayer was stained with PE-streptavidin and imaged.

### Neutrophil transmigration assay

HUVECs were plated onto type I collagen-coated polycarbonate inserts (transwell inserts, 6.5-mm diameter, 3-μm pore for 24-well plates; Costar, MA). HUVECs (10^5^ cells) were grown in 300 μL of EGM-2. An additional 500 μL of complete medium was added to the lower chamber of each well, and the HUVECs were grown to confluence over 24 h. Isolated PMNs (10^6^ cells) were activated with TNF-α (20 ng/mL, 30 min, 37 °C), pre-treated with LA-1 or DMSO, or were left untreated and were added to the upper chamber of the transwells. The lower well contained 500 μL of EBM-2 with 10^−6^ M fMLP as a chemoattractant. After 3 h at 37 °C in 5% CO_2_, the migrated cells were counted. Experiments were performed in triplicate.

### Time-lapse video microscopy

Neutrophils from healthy donors and ICU patients were allowed to crawl on activated HUVEC monolayers to determine the effect of LA-1 on neutrophil migration dynamics. HUVEC monolayers were grown on delta T dishes coated with 40 μg/mL type I collagen and TNF-α-activated. Neutrophils were either given no treatment, 15 μM LA-1, or DMSO vehicle control. Cell interactions were allowed to adhere at 37 °C, and neutrophil migration dynamics were imaged. × 20 bright-field images were captured every 30 s for 20 min. Neutrophil migration paths were tracked using a manual tracking software which is an ImageJ plug-in available from ibidi. Videos were uploaded into ImageJ, and for a given cell, the centroid was identified. At periodic intervals, the *x*, *y* coordinates were recorded and processed by the plug-in to provide path length, distance, and speed.

### Data analysis

Data are reported as mean normalized monolayer resistance ± standard error of the mean, with *n* being the number of independent experiments. Values are normalized to initial baseline resistance (observed resistance/initial resistance). Barrier function assays were evaluated using three-way or two-way analysis of variance with post hoc Tukey analysis as appropriate. Statistical significance was defined as *p* < 0.05.

## Results

### Neutrophils from critically ill trauma patients show significant endothelial barrier disruption

**Fig. 1:**
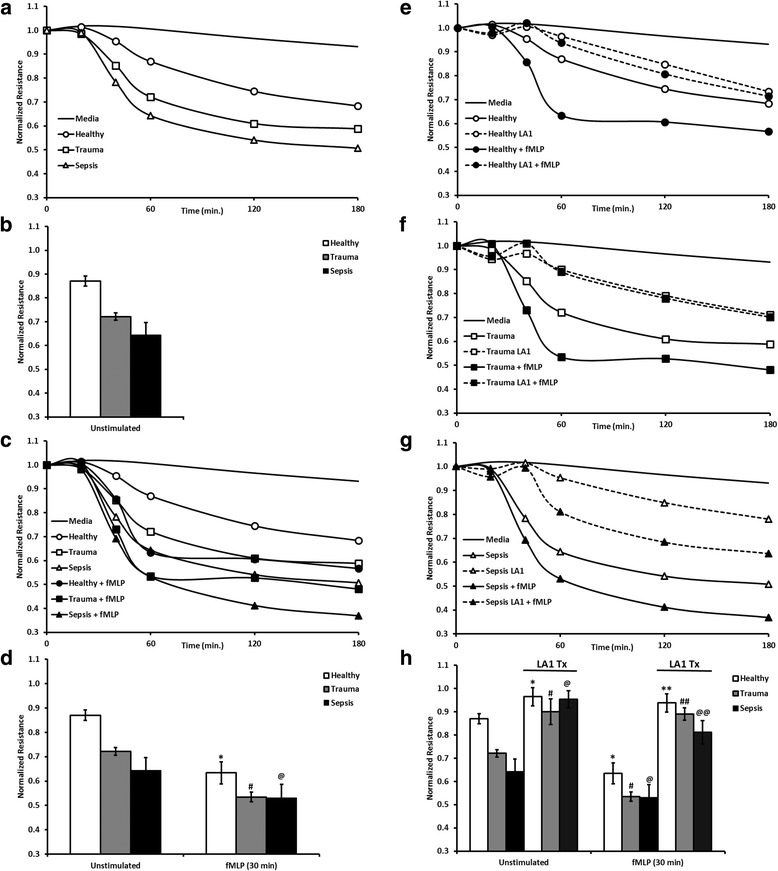
Endothelial barrier disruption by neutrophils isolated from healthy (*n* = 14), trauma (*n* = 8), and septic donors (*n* = 6) and protection by LA-1. Change in normalized resistance with respect to time. *T* = 0 represents time of neutrophil addition to HUVECs activated by TNF for 4 h. **a** Representative tracing of electrical resistance across activated HUVEC monolayers in the presence of neutrophils obtained from healthy, trauma, or septic donors. **b** Values are mean ± SEM of normalized resistance at 60 min following PMN addition. Analyses were performed evaluating the effect of neutrophils obtained from critically ill trauma patients (*n* = 8), from critically ill septic patients (*n* = 6), and from healthy controls (*n* = 14) and assayed in duplicate on each experimental day. **c** Representative ECIS tracing of neutrophils added to HUVEC monolayer 30 min after fMLP (10^−6^ M). **d** Values are mean ± SEM of normalized resistance at 60 min following PMN addition ± fMLP. **e**–**g** Representative tracings of neutrophils ± fMLP activation in the presence or absence of LA-1. **e** Neutrophils obtained from healthy donors. **f** Neutrophils obtained from trauma patients. **g** Neutrophils obtained from septic patients. **h** Values are mean ± SEM of normalized resistance at 60 min following PMN addition ± fMLP. *p < 0.05 vs. healthy unstimulated; **p < 0.05 vs. healthy fMLP-stimulated; ^#^*p* < 0.05 vs. trauma unstimulated; ^##^*p* < 0.05 vs. trauma fMLP-stimulated; ^@^*p* < 0.05 vs. septic unstimulated; and ^@@^*p* < 0.05 vs. septic fMLP-stimulated. Statistical significance determined (*p* < 0.05) using three-way ANOVA with post hoc Tukey analysis

The ECIS system uses changes in transendothelial resistance as a measure of endothelial barrier integrity with diminished resistance correlating with loss of barrier function upon challenge. Prior work from our laboratory showed that neutrophils induced greater loss of barrier function when human umbilical cord endothelial cells (HUVECs) were TNF-α-treated as compared to untreated HUVECs demonstrating the expected effectiveness of TNF-α activation [12]. Accordingly, TNF-α-activated HUVECs were used throughout the work shown below to mimic the systemic cytokine response during injury and/or sepsis, and thrombin served as a positive control for loss of barrier function. We reported previously that the extent to which neutrophils obtained from septic patients induced endothelial barrier disruption, as measured by ECIS, exceeded that of cells from healthy donors and was similar to cells from healthy donors following fMLP activation [12]. In the current study, neutrophils were obtained from injured but non-septic patients to determine if trauma elicits systemic neutrophil activation with regard to causing barrier damage. Analyses were performed evaluating the effect of neutrophils obtained from critically ill trauma patients (*n* = 8), from critically ill septic patients (*n* = 6), and from healthy controls (*n* = 14). Figure 1a shows representative ECIS tracings to illustrate the information obtained from this technology regarding the kinetics and magnitude of endothelial barrier loss. As noted from the tracing, significant neutrophil damage to barrier function is observed 60 min after addition of these cells to the endothelial monolayer. Given the magnitude of the effect, this time point was selected for statistical analysis to demonstrate endothelial damage from neutrophils obtained from multiple donors (Fig. 1b) and to assess the therapeutic efficacy of LA-1. Since time is not a variable, statistical analysis was performed using a three-way mixed ANOVA. All neutrophil populations caused a loss of endothelial barrier integrity, with the neutrophils isolated from healthy volunteers causing the least amount of damage (0.87 ± 0.02 at 60 min), as compared to those from trauma patients or septic patients (0.72 ± 0.05 and 0.64 ± 0.06, respectively, at 60 min) or those from healthy neutrophils stimulated ex vivo with fMLP (0.63 ± 0.05 at 60 min).

### Neutrophils from trauma patients are subject to a second hit

Given that critically ill patients are at risk for a second insult, whether it be a surgery or a secondary infection, we sought to determine if the neutrophils from trauma patients could be further stimulated to cause greater endothelial barrier disruption. Patient neutrophils were allowed to tether to the endothelial monolayer for 30 min and were then exposed to an activating dose of fMLP (10^−6^ M) (Table 1). As seen in Fig. 1c, d, fMLP stimulation increased barrier damage from PMNs obtained from healthy donors (0.87 ± 0.02 vs. 0.63 ± 0.05; unstimulated vs. fMLP-stimulated, *p < 0.05), trauma patients (0.72 ± 0.05 vs. 0.53 ± 0.06; unstimulated vs. fMLP-stimulated, *p < 0.05), or septic patients (0.64 ± 0.06 vs. 0.53 ± 0.03; unstimulated vs. fMLP-stimulated, *p* < 0.05).

**Table 1:** Characteristics of critically ill patients

Sepsis vs. trauma	Sepsis (*n* = 6)	Trauma (*n* = 8)	*P* value
Age (mean ± SD)	74.7 ± 11.5	48.0 ± 21.6	0.0185
Male sex (%)	33.3%	62.5%	0.35
Source of sepsis (%)			
Abdominal	4	NA	NA
Other	2		
ISS		20.1 ± 9.5	
White cell count (× 10^9^ cells/L) (mean ± SD)	22.38 ± 9.9	18.4 ± 5.2	0.35
Neutrophil count (×10^9^ cells/L) (mean ± SD)	19.17 ± 8.7	13.94 ± 4.3	0.17
APACHE II Score (mean ± SD)	18.17 ± 4.9	10.25 ± 5.17	0.0152
SOFA Score (mean ± SD)	5.17 ± 2.2	2.63 ± 2.2	0.0583

Characteristics listed are the highest values in the first 24 h from admission

### Treatment of neutrophils with leukadherin-1 reduces loss of barrier integrity caused by neutrophils

Using rodent models of inflammation, LA-1 therapy showed a reduction in number of neutrophils extravasated into the site of inflammation with transiently increased local adhesion along the vasculature [10]. This transiently increased adhesion allows for the potential for LA-1 to exacerbate neutrophil-induced endothelial damage by prolonging the close contact between neutrophils and endothelial cells. For this reason, we determined the effect of LA-1 on barrier function with neutrophils isolated from healthy, trauma, or septic patients. Neutrophils treated with LA-1 caused significantly less barrier disruption at 60 min than untreated neutrophils isolated from healthy (vs. 0.87 ± 0.02 vs. 0.96 ± 0.02; untreated vs. LA-1-treated, *p < 0.05, Fig. 1e, h), trauma (0.72 ± 0.05 vs. 0.90 ± 0.04, untreated vs. LA-1-treated, ^#^*p* < 0.05, Fig. 1f, h), or septic donors (0.64 ± 0.06 vs. 0.95 ± 0.04; untreated vs. LA-1-treated, ^@^*p* < 0.05, Fig. 1g, h). LA-1 pretreatment also decreased the amount of damage caused by fMLP-stimulated neutrophils (30 min stimulation) from healthy volunteers (0.063 ± 0.05 vs. 0.94 ± 0.02, untreated vs. LA-1-treated, **p < 0.05, Fig. 1e, h), trauma patients (0.53 ± 0.06 vs. 0.89 ± 0.04; untreated vs. LA-1-treated, ^##^*p* < 0.05, Fig. 1f, h), and sepsis patients (0.53 ± 0.03 vs. 0.81 ± 0.05; untreated vs. LA-1-treated), ^@@^*p* < 0.05, Fig. 1g, h). The decrease in endothelial damage for patient samples pretreated with LA-1 brought it to levels indistinguishable from neutrophils from healthy volunteers. In the absence of neutrophils, LA-1 had no effect on HUVEC barrier function (data not shown). LA-1 alone does not affect neutrophil viability and is non-toxic [10]. These findings show the potency of LA-1 in that it can afford barrier protection even in the presence of neutrophils following secondary stimulation.

### Leukadherin-1 reduces endothelial permeability and underlying collagen exposure

**Fig. 2:**
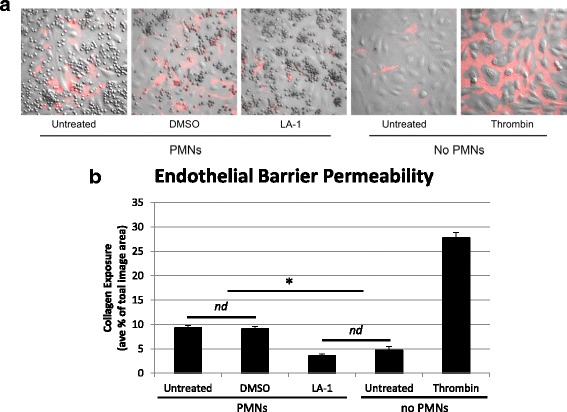
Visualization of neutrophil-induced permeability of confluent TNF-activated HUVEC monolayers and protection by LA-1. Neutrophils from healthy donors were activated by fMLP prior to addition to endothelial monolayers grown on biotinylated type I collagen as described in the “Methods” section. Localized permeability visualized by PE-strepavidin and imaged. **a** fMLP-activated neutrophils were left untreated, treated with DMSO as vehicle control or 15 μM LA-1. Baseline permeability is shown by the absence of PMNs, and agonist-induced permeability by thrombin are shown as technical controls. **b** Quantification of collagen exposure as % total area. Values are mean ± SEM from three identical experiments in duplicates. Two-way ANOVA with post hoc Tukey analysis, *p < 0.05 LA-1 vs. untreated or DMSO-treated neutrophils

In a complementary technical approach to demonstrate the effect of LA-1 on endothelial integrity, HUVECs were plated on biotinylated collagen-coated delta T dishes and then stained with streptavidin-PE to identify specific regions of matrix exposure (Fig. 2a). Neutrophils from healthy volunteers were either pretreated with LA-1 and DMSO or left untreated and were incubated on the TNF-α-activated endothelium for 40 min after fMLP (10^−6^ M) stimulation. Unchallenged HUVECs or HUVECs treated with thrombin to induce permeability served as technical controls for the system. Activated neutrophils treated with LA-1 caused significantly less collagen exposure than the untreated or vehicle control neutrophils (Fig. 2b) consistent with findings using ECIS (Fig. 1) in demonstrating the ability of LA-1 to protect an endothelial monolayer from damage from inflammatory neutrophils.

### Treatment of neutrophils with leukadherin-1 reduces neutrophil transmigration

**Fig. 3:**
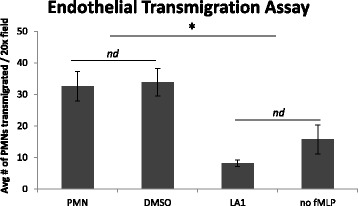
LA-1 inhibits neutrophil transendothelial migration. Neutrophils from healthy donors were activated by fMLP prior to addition onto endothelial monolayers grown on type I collagen as described in the “Methods” section. PMNs migrated across endothelial were counted, and data is shown as mean ± SEM, two-way ANOVA with post hoc Tukey analysis, *p < 0.05 LA-1 vs. untreated or DMSO-treated neutrophils

Experiments were conducted to determine if LA-1 could dampen diapedesis of activated neutrophils through HUVEC monolayers. TNF-α-activated neutrophils from healthy volunteers (*n* = 3) were either given no treatment, 15 μM LA-1, or DMSO vehicle control. fMLP (10^−6^ M) was either added to the lower wells as a chemoattractant, or not for control, and after 1 h the number of neutrophils that had transmigrated to the bottom well were counted. As shown in Fig. 3, LA-1 significantly reduced the number of neutrophils that transmigrated.

### Treatment of neutrophils with leukadherin-1 reduces neutrophil motility on an endothelial layer

**Fig. 4:**
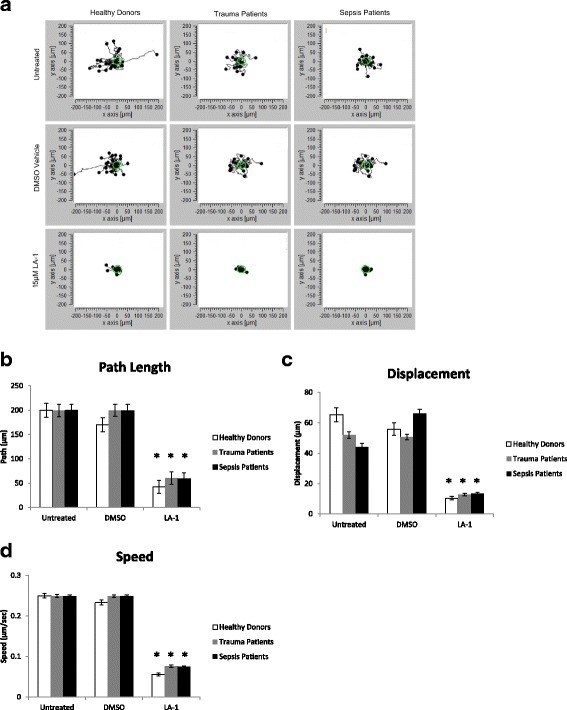
LA-1 inhibits migration of neutrophils on endothelial monolayers. HUVEC monolayers were grown on delta T dishes coated with 40 μg/mL type I collagen and TNF-α activated. Neutrophils were either TNF-α-activated and given no treatment, 15 μM LA-1, or DMSO vehicle control. Cell interactions were allowed to adhere at 37 °C and neutrophil migration dynamics were imaged. × 20 bright-field images were captured every 30 s for 20 min. Neutrophil migration paths were tracked in ImageJ and analyzed using ibidi’s Chemotaxis and Migration Tool (**a**). LA-1 treatment reduced neutrophil path length (**b**), displacement (**c**), and speed (**d**) regardless of donor. Two-way ANOVA with post hoc Tukey analysis, *p < 0.05 LA-1 vs. untreated or DMSO-treated neutrophils

Neutrophils tether to and migrate on an endothelial monolayer prior to diapedesis. Experiments were conducted to test the hypothesis that the migration of human neutrophils on an endothelial monolayer was affected by LA-1. Neutrophils on TNF-α-activated HUVEC monolayers were stimulated with fMLP (10^−6^ M) and exposed to either no treatment, 15 μM LA-1, or DMSO vehicle control to assess the impact of LA-1 on cell motility. Neutrophil migration paths were tracked and analyzed. LA-1 pre-treated neutrophils exhibited a decrease in movement compared to untreated healthy, trauma, or sepsis neutrophils (Fig. 4a, Additional files 1 and 2). Regardless of donor status, the LA-1 pre-treated neutrophils had a decreased path length (Fig. 4b), decreased displacement (Fig. 4c), and decreased speed (Fig. 4d). Given that neutrophil migration on an endothelial monolayer is in part driven by leukocyte integrins, that migratory behaviour is altered by LA-1 is fully consistent with an integrin-based mechanism of action.

## Discussion

Leukocyte integrins including CR3 mediate the essential steps through which blood leukocytes emigrate into a site of tissue injury or inflammation. Blocking the function of integrins is a logical approach towards the development of anti-inflammatory agents designed to treat disease states where inflammation is excessive and contributes to pathology rather than host defense. LA-1 offers an alternative therapeutic approach towards control of inflammatory-based diseases by activating rather than blocking CR3. A pharmacological rationale underlies the development of an agonist as compared to a blocking compound in that blockade necessitates neutralization of a predominance of cell surface receptors to be effective while it has been shown that integrin-regulated cellular function need only include a subpopulation of receptors held in the activated state. LA-1 is a specific agonist of the integrin CR3 and has proven to be easily delivered in vivo and reduce the neutrophilic infiltrate sites of infection or injury in animal models [10, 14]. It is proposed that LA-1 enhances CR3 binding to endothelial ICAM and tether blood neutrophils to the vasculature such that tissue emigration does not ensue. A reasonable concern in the development of LA-1 for clinical use is that an agent that promotes neutrophil-endothelial binding may exacerbate neutrophil-mediated vascular damage. The essential objective of the current study was to determine if LA-1 contributes to additional loss of endothelial barrier function than that caused by activated human neutrophils alone. Endothelial monolayers were challenged by neutrophils activated ex vivo or obtained from septic or trauma patients with and without a second activation stimulant to maximize the damaging capacity of the neutrophil. In no case did the presence of LA-1 worsen endothelial damage, and surprisingly, in all cases, protection of barrier function was afforded. Findings using an initial, small cohort of patients provide a proof-of-principle that development of LA-1 for patient use is not likely to be confounded by agonist-induced vascular damage. These preliminary findings justify further studies using neutrophils obtained from a larger cohort of trauma and septic patients to better correlate the state of critical illness with efficacy of LA-1 treatment.

LA-1 is proposed to increase adhesion of peripheral blood neutrophils to vascular endothelial cells and, in so doing, reduce neutrophil entry into the inflammatory site [9, 15]. However, LA-1 has not been evaluated specifically for its effect on the barrier function of an endothelial monolayer challenged with human neutrophils obtained from ICU patients with active inflammatory disease. Here, we tested the hypothesis that prolonged neutrophil-endothelial engagement by LA-1 would result in compromised barrier function as compared to cells without LA-1 treatment. Surprisingly, and through a mechanism yet to be elucidated, LA-1 treatment preserved barrier function in neutrophils from trauma and septic donors even following a second inflammatory challenge with fMLP to maximize the activation state of the neutrophil. These experiments were quantified by endothelial resistance measures obtained in real time by ECIS. For an added proof-of-principle that LA-1 protects monolayer integrity, we used a collagen exposure assay where activated neutrophils caused focal points of permeability without LA-1 and which were significantly diminished in area upon LA-1 treatment. Additional experiments showed that LA-1-treated neutrophil-endothelial co-cultures reduced the migration of neutrophils on and through the monolayer.

In vivo, LA-1 showed a reduction in the infiltration of neutrophils in several rodent models of sterile inflammation including thioglycolate-induced peritonitis, TNF-α-injected cremaster muscle, arterial balloon injury, bile duct injury, and kidney allograft rejection [14–16]. LA-1 improved survival in a cecal ligation and puncture model with corresponding decrease in serum proinflammatory cytokines and decreased bacterial count in the peritoneum [17]. Moreover, when isolated murine or human macrophages were treated with LPS or LPS plus LA-1, the agonist suppressed TLR signaling via the AKT/FOXO3/IRF3/7 axis in a CD11b-dependent fashion [17]. In mouse macrophages, activated CD11b negatively regulates TLR signaling such that a CR3 deficiency resulted in enhanced TLR-mediated responses and increased susceptibility to endotoxemia and sepsis [18]. Mechanistically, it was shown that CR3 activation causes ubiquitinylation/degradation of MyD88 and TRIF in the presence of TLR activation such that CR3 keeps a TLR response in check. CR3-deficient mice injected with TLR ligands had increased NFκB transcription and upregulated interferon pathways with increased IL-6, TNF-α, and IFN-β. Therefore, by virtue of targeting CR3, LA-1 is anti-inflammatory in the presence of TLR activation in vitro and in vivo.

In SLE, LA-1 has found a therapeutic role in restoring CR3 function. A mutant CR3 variant that results in a substitution of arginine for a histidine at position 77 (R77H) encoded by the rs1143679 polymorphism is strongly associated with SLE. This variant keeps CR3 in the inactive state that in turn may limit ligand affinity and adhesive functions [19]. Because CR3 in these patients cannot become activated, it cannot control TLR signaling and they have basal levels of type I interferon and IRF7 and decreased FOXO3 [17]. It may follow that the inability to activate CR3 contributes to the autoimmune state. LA-1 is able to convert the resting state of CR3 in macrophages derived from carriers of CD11B SNP to an activated state and suppresses unregulated TLR-dependent inflammation [20].

Therapeutics designed to block or antagonize the function of a target receptor, including integrins, have the challenge of needing to inhibit the majority of that receptor expressed on the cell surface in order to maintain a sufficient level of inhibition to achieve pharmacological efficacy. Instead, LA-1 functions as an integrin agonist and is now understood to stabilize the leukocyte integrin CR3 in an intermediate state of activation. It has also shown favorable pharmacodynamics in animal models of inflammation. Therefore, integrin activation may provide a new effector mechanism to control inflammatory diseases. We now show that LA-1 protects barrier function even though contact and motility along a monolayer show increased tethering of neutrophils. That barrier function is protected even though contact is pronounced by drug is surprising and counterintuitive but supports the benefit and safety in considering the anti-inflammatory application of LA-1 in the hyperinflammed trauma or septic patient. That the drug operates in vascular protection when challenged by cells from ICU donors even after secondary ex vivo activation widens its potential therapeutic patient population.

## Conclusions

Leukadherin-1 represents a novel class of drug indicated to dampen tissue damage caused by neutrophil activation. Prior reports demonstrate that LA-1functions as an integrin agonist and promotes, rather than lessens, neutrophil-endothelial binding. Data in this report demonstrate that LA-1 does not increase neutrophil damage of endothelial function as might be anticipated by an integrin agonist and, indeed, findings show LA-1-mediated protection of endothelial barrier by activated neutrophils. Given the need for new therapeutic modalities to treat the hyperinflammatory sequelae that follow trauma as well as sepsis, a close examination of the effect of experimental drugs on endothelial barrier function is warranted.

## Key messages

- Neutrophils from trauma patients mediate endothelial barrier loss to the same quantitative extent as cells obtained from septic donors
- Neutrophils from trauma patients are subject to secondary stimulation by fMLP resulting in augmented compromise of endothelial barrier function.
- Regardless of whether neutrophils are obtained from trauma or septic patients or subject to secondary fMLP stimulation, LA-1 significantly protected endothelial barrier function from neutrophilic damage.

## Additional files

Additional file 1: Representative video of fMLP-activated human neutrophils migrating on TNF-α-stimulated HUVEC monolayer for 30 min. DMSO added as vehicle control. (AVI 1246 kb)

Additional file 2: Representative video of fMLP-activated human neutrophils migrating on TNF-α-stimulated HUVEC monolayer for 30 min in the presence of 15 μM LA-1. (AVI 1511 kb)

## Abbreviations

ANOVA: Analysis of variance
ECIS: Electric cell-substrate impedance sensing
fMLP: n-Formyl-l-methionyl-l-leucyl-l-phenylalanine
HUVEC: Human umbilical vein endothelial cells
LA-1: Leukadherin-1
TNF-α: Tissue necrosis factor-α

## References

[1] Ley K, Laudanna C, Cybulsky MI, Nourshargh S (2007). Getting to the site of inflammation: the leukocyte adhesion cascade updated. Nat Rev Immunol.

[2] Nathan C, Ding A (2010). Nonresolving inflammation. Cell.

[3] Brown KA, Brain SD, Pearson JD, Edgeworth JD, Lewis SM, Treacher DF (2006). Neutrophils in development of multiple organ failure in sepsis. Lancet.

[4] Kumar P, Shen Q, Pivetti CD, Lee ES, Wu MH, Yuan SY (2009). Molecular mechanisms of endothelial hyperpermeability: implications in inflammation. Expert Rev Mol Med.

[5] Crouser ED, Matthay MA (2017). Endothelial damage during septic shock: significance and implications for future therapies. Chest.

[6] Phillipson M, Heit B, Colarusso P, Liu L, Ballantyne CM, Kubes P (2006). Intraluminal crawling of neutrophils to emigration sites: a molecularly distinct process from adhesion in the recruitment cascade. J Exp Med.

[7] Herter J, Zarbock A (2013). Integrin regulation during leukocyte recruitment. J Immunol.

[8] Harlan JM, Winn RK (2002). Leukocyte-endothelial interactions: clinical trials of anti-adhesion therapy. Crit Care Med.

[9] Celik E, Faridi MH, Kumar V, Deep S, Moy VT, Gupta V (2013). Agonist leukadherin-1 increases CD11b/CD18-dependent adhesion via membrane tethers. Biophys J.

[10] Maiguel D, Faridi MH, Wei C, Kuwano Y, Balla KM, Hernandez D, Barth CJ, Lugo G, Donnelly M, Nayer A (2011). Small molecule-mediated activation of the integrin CD11b/CD18 reduces inflammatory disease. Sci Signal.

[11] Tsikitis VL, Morin NA, Harrington EO, Albina JE, Reichner JS (2004). The lectin-like domain of complement receptor 3 protects endothelial barrier function from activated neutrophils. J Immunol.

[12] Fox ED, Heffernan DS, Cioffi WG, Reichner JS (2013). Neutrophils from critically ill septic patients mediate profound loss of endothelial barrier integrity. Crit Care.

[13] Dubrovskyi O, Birukova AA, Birukov KG (2013). Measurement of local permeability at subcellular level in cell models of agonist- and ventilator-induced lung injury. Lab Investig.

[14] Khan SQ, Guo L, Cimbaluk DJ, Elshabrawy H, Faridi MH, Jolly M, George JF, Agarwal A, Gupta V (2014). A small molecule beta2 integrin agonist improves chronic kidney allograft survival by reducing leukocyte recruitment and accompanying vasculopathy. Front Med.

[15] Faridi MH, Altintas MM, Gomez C, Duque JC, Vazquez-Padron RI, Gupta V (2013). Small molecule agonists of integrin CD11b/CD18 do not induce global conformational changes and are significantly better than activating antibodies in reducing vascular injury. Biochim Biophys Acta.

[16] Joshi N, Kopec AK, Ray JL, Cline-Fedewa H, Nawabi A, Schmitt T, Nault R, Zacharewski TR, Rockwell CE, Flick MJ (2016). Fibrin deposition following bile duct injury limits fibrosis through an alphaMbeta2-dependent mechanism. Blood.

[17] Faridi MH, Khan SQ, Zhao W, Lee HW, Altintas MM, Zhang K, Kumar V, Armstrong AR, Carmona-Rivera C, Dorschner JM (2017). CD11b activation suppresses TLR-dependent inflammation and autoimmunity in systemic lupus erythematosus. J Clin Invest.

[18] Han C, Jin J, Xu S, Liu H, Li N, Cao X (2010). Integrin CD11b negatively regulates TLR-triggered inflammatory responses by activating Syk and promoting degradation of MyD88 and TRIF via Cbl-b. Nat Immunol.

[19] Rosetti F, Chen Y, Sen M, Thayer E, Azcutia V, Herter JM, Luscinskas FW, Cullere X, Zhu C, Mayadas TN. A lupus-associated Mac-1 variant has defects in integrin allostery and interaction with ligands under force. Cell Rep. 2015;10(10):1655–64. 10.1016/j.celrep.2015.02.037 PMC4567551 25772353

[20] Roberts AL, Furnrohr BG, Vyse TJ, Rhodes B (2016). The complement receptor 3 (CD11b/CD18) agonist Leukadherin-1 suppresses human innate inflammatory signalling. Clin Exp Immunol.

